# Bald thigh syndrome in sighthounds—Revisiting the cause of a well-known disease

**DOI:** 10.1371/journal.pone.0212645

**Published:** 2019-02-22

**Authors:** Magdalena A. T. Brunner, Silvia Rüfenacht, Anina Bauer, Susanne Erpel, Natasha Buchs, Sophie Braga-Lagache, Manfred Heller, Tosso Leeb, Vidhya Jagannathan, Dominique J. Wiener, Monika M. Welle

**Affiliations:** 1 Institute of Animal Pathology, Vetsuisse Faculty, University of Bern, Bern, Switzerland; 2 DermFocus, University of Bern, Bern, Switzerland; 3 DermaVet, Tierklinik Aarau West, Oberentfelden, Switzerland; 4 Institute of Genetics, Vetsuisse Faculty, University of Bern, Bern, Switzerland; 5 Nano Imaging Lab, SNI, University of Basel, Basel, Switzerland; 6 Department for BioMedical Research (DBMR), University of Bern, Bern, Switzerland; 7 Department of Veterinary Pathobiology, Texas A&M University, College Station, United States of America; INSERM, FRANCE

## Abstract

Bald thigh syndrome is a common hair loss disorder in sighthounds. Numerous possible causes, including environmental conditions, trauma, stress, endocrinopathies and genetic components have been proposed, but only endocrinopathies have been ruled out scientifically. The overall goal of our study was to identify the cause of bald thigh syndrome and the pathological changes associated with it. We approached this aim by comparing skin biopsies and hair shafts of affected and control dogs microscopically as well as by applying high-throughput technologies such as genomics, transcriptomics and proteomics. While the histology is rather unspecific in most cases, trichogram analysis and scanning electron microscopy revealed severe structural abnormalities in hair shafts of affected dogs. This finding is supported by the results of the transcriptomic and proteomic profiling where genes and proteins important for differentiation of the inner root sheath and the assembly of a proper hair shaft were downregulated. Transcriptome profiling revealed a downregulation of genes encoding 23 hair shaft keratins and 51 keratin associated proteins, as well as desmosomal cadherins and several actors of the BMP signaling pathway which is important for hair shaft differentiation. The lower expression of keratin 71 and desmocollin 2 on the mRNA level in skin biopsies corresponded with a decreased protein expression in the hair shafts of affected dogs. The genetic analysis revealed a missense variant in the *IGFBP5* gene homozygous in all available Greyhounds and other sighthounds. Further research is required to clarify whether the *IGFBP5* variant represents a predisposing genetic risk factor. We conclude from our results that structural defects in the hair shafts are the cause for this well-known disease and these defects are associated with a downregulation of genes and proteins essential for hair shaft formation. Our data add important knowledge to further understand the molecular mechanisms of HF morphogenesis and alopecia in dogs.

## Introduction

One of the most characteristic features of hair follicles (HFs) is their self-renewal throughout the entire life of an individual to continuously produce new hair shafts (HSs). The self-renewal process is organized in the hair cycle during which the HF undergoes periodic stages of growth (anagen), regression (catagen), and quiescence (telogen) [1, 2]. The HS elongates during late anagen by the division of lineage-restricted matrix progenitors. These cells divide and migrate upward while differentiating to trichocytes. During differentiation, the nuclear function of the cells ceases, the non-keratin cell components break down and the synthesis of keratins and keratin-associated proteins (KAPs) increases. The precipitation of keratins and the loss of water finally results in terminal cornification, associated with hardening of the HS. During this process cell membranes of neighbouring trichocytes become more closely apposed and the number of cell junction complexes becomes higher, indicating an increased cellular adhesion and intercellular communication. At the level of the proximal end of the isthmus the mature HS detaches from the IRS (Adamson`s fringe) [3].

The individual trichocyte is composed of several macrofibrils, which are linked together by desmosomes containing several adhesion molecules such as desmoplakin and desmoglein. Macrofibrils in turn are composed of numerous intermediate filaments (IFs) embedded in an amorphous matrix composed of KAPs. An extensive network of inter- and intramolecular protein-protein crosslinks the IFs to the KAPs. The IFs are formed by a complex interaction of acidic type I and basic type II keratins [4, 5]. While the IFs provide the HS with a high tensile strength the matrix is important for the toughness, pliability, and resistance against microbial attacks [6]. Chemically, the HS is composed of 65–90% proteins and, depending on the humidity, up to 32% water. In addition, it contains lipids, pigments, and metals [7]. The main proteins of the trichocytes are keratins and KAPs. Hair keratins differ from epithelial keratins in their high content in cysteine residues used for the formation of inter- and intra-molecular disulfide bonds during the keratinization process [8]. Different keratins are expressed in the different regions of the HS. In humans, the medulla and cortex contain twelve different hair keratins, whereas only six different keratins are expressed in the cuticle. Furthermore, a high number of epithelial keratins are expressed in the medulla [9–11]. KAPs are classified by their amino acid composition into high sulfur, ultra-high sulfur and tyrosine/glycine rich KAPs [12]. Like keratins, KAPs are differentially expressed within the HS. In human hair, up to 19 high sulphur, 24 ultra-high sulphur, and 14 high-glycine tyrosine KAPs have been found in the cortex, whereas 22, 34, and nine different KAPs, respectively have been found in sheep [12]. The KAPs are encoded by a large multigene family of which 92 protein coding genes are currently known in humans [13, 14].

In cross section, the HS is composed of three different layers: the cuticle, the cortex and the medulla. The cuticle is the outermost, protective layer and consists of several layers of flat, thin overlapping trichocytes that are arranged like shingles on a roof. The cuticle cells undergo flattening as they emerge from their progenitors in the bulb. The distance of the cuticle cells from each other and the morphology varies between dog breeds and accounts for the smoothness of the hair and the tendency to felt [4, 15]. The cuticle is affected by environmental conditions such as harsh weather conditions and brushing [16]. The cortex accounts for the major mass of the hair fiber and is important for the tensile properties of the HS. It is built of trichocytes that change in shape from spherical close to the bulb to highly elongated and aligned with the long axis of the HS in the more distal parts. The medulla is the innermost layer of the HS and consists of a column of large, loosely packed and vacuolated cells with a high nuclear–cytoplasmic ratio. The cells are horizontally oriented and randomly staggered. The medulla can be very prominent in some species with coarse hair or can be absent in fine animal and human scalp hairs [12, 15, 17].

Bald thigh syndrome (BTS) is a hair loss disorder seen in Greyhounds and other sighthound breeds into which the Greyhound has been introgressed such as Whippets, Galgo Español, and Magyar Agár. BTS is characterized by bilateral hair loss on the caudal and lateral thighs, but alopecia may extend to the distal hind legs, the ventral abdomen and the chest. In some cases even the ventral neck is involved. Dogs of any age and sex may be affected. No scientific information is available under which circumstances the hair loss occurs but anecdotal information received from owners, breeders and internet forums indicate that the hair loss mostly starts when dogs are adult. It is frequently reported that hair loss begins when dogs start their training on the race track but it is also seen in dogs that are solely kept as companion dogs. Alopecia may wax and wane and no relation to stress or food can be made. It has been reported that up to 16% of the Greyhounds in long term training are affected [18]. The cause of BTS is unclear but numerous possible causes have been proposed. The alopecia has been associated with dogs rubbing against the sides of their crates, training methods, physical stress, diet, environmental conditions, estrous cycle and also an over-production of cortisol induced by a rigorous training program. In addition, a genetic component is suspected since BTS affects only Greyhounds and related breeds. In veterinary textbooks this syndrome is reported as pattern baldness [19, 20]. In the only scientific publication about this syndrome it was hypothesized that BTS is associated with hypothyroidism or hyperadrenocorticism. However, neither morphological changes in the adrenal glands, thyroid glands or the skin confirmed this hypothesis. Histological findings reported in this publication were not uniform in all Greyhounds with BTS. Common changes included dilatation of follicular infundibula, which often contained keratin and HSs and the presence of catagen HFs [21].

The overall goal of our study was to identify the cause of BTS and pathological changes associated with it. We addressed this by 1) histological evaluation of skin biopsies from Greyhounds with BTS in comparison to skin biopsies from haired Greyhounds, 2) investigation of the HS structure using trichograms and scanning electron microscopy of affected and control Greyhounds and Whippets, 3) transcriptome analysis of skin biopsies from affected and control Greyhounds to identify differences in gene expression in order to gain insight into the molecular mechanisms which may be involved in the pathogenesis of BTS, 4) assessment of the protein composition of HSs of affected and control dogs, and 5) comparison of whole genome sequence data from affected and unaffected Greyhounds in comparison to genome sequence data of dogs from other breeds that are not predisposed to BTS.

## Material and methods

### Ethics statement

All hair samples, EDTA blood and skin biopsies were taken with informed owner consent and with the permission of the cantonal animal welfare committee, Switzerland (permission number BE38/17). Permission of the cantonal animal welfare committee was given prospectively and comprises the intravenous withdrawal of blood and taking skin biopsies in dogs which have been identified by veterinarians to be suitable for the study. Biopsies were taken under local anesthesia using either lidocaine 2% or ropivacain 2%. In a few cases sedation was necessary. The dogs were sedated after 5 hours of fasting with medetomidine (5-10mcg/kg IM) and antagonised intramuscularely (IM) with the same volume of atepamizole as was given with medetomidine.

### Skin biopsies

Skin biopsies were taken from seven Greyhounds with BTS, six control Greyhounds and one Magyar Agar. All dogs were privately owned and kept as pets. In addition, four archival biopsies from Greyhounds with BTS and six biopsies from control Greyhounds that were submitted to the University of Bern for routine diagnostic purposes were evaluated histologically. The diagnosis BTS was based on the clinical phenotype of the dogs by a board certified veterinary dermatologist (SR). Endocrinopathies were excluded by the absence of systemic signs and appropriate laboratory tests. From each dog two 6 mm punch biopsies were taken from the caudal thigh. In dogs with BTS the biopsies were taken in the alopecic area. From each dog one biopsy was immediately put in RNA*later* (76106; Qiagen) and frozen at -80°C prior to RNA extraction. The other biopsy was fixed in 10% buffered formalin, processed routinely and stained with hematoxylin and eosin prior to the histological evaluation (S1 Table).

### Histological analysis

We evaluated skin biopsies sampled for this study and archival biopsies from 11 Greyhounds with BTS and 13 controls (12 Greyhounds, 1 Magyar Agar), respectively. The formalin fixed biopsies were embedded in paraffin, cut as 4 μm section and stained with hematoxylin and eosin using standard procedures. The histological evaluation was performed blinded by two experienced dermatopathologists (DJW and MMW) and special attention was payed to the density, size and cycle stages of the HFs and the diameter of the HSs.

### Trichogram analysis

Trichograms were assessed from dogs with BTS (Greyhounds, n = 6; Whippets, n = 10) and controls dogs (Greyhounds, n = 5; Whippets n = 12; Magyar Agar, n = 1) (S1 and S2 Tables).

To analyze HS quality, 10–70 HSs were plucked with a mosquito clamp from the border of the alopecic thigh and the haired back of affected dogs. In the alopecic areas only very few HSs were visible and these were plucked as well. A similar number of hairs were also plucked from the haired thigh and the back of control dogs. The hairs were placed on a glass slide, cleaned with two drops of chloral-lactophenol, covered with a cover slip and examined with a 200x magnification under the microscope. The hair cycle stage and the quality of the HSs were recorded.

Statistics were performed by assessing first normality of the data with the Shapiro test. Thereafter, the Wilcoxon signed rank test was used to assess the difference in the percentage of fractured HSs plucked from 1) the thighs of affected vs thighs of control dogs, 2) the back of affected dogs vs the back of control dogs, 3) the thighs of affected dogs vs the back of control dogs and 4) the back of affected dogs vs the thighs of control dogs. The paired samples Wilcoxon test was used to compare the percentage of fractured HSs plucked from 1) the thigh vs the back of affected dogs and 2) the thigh vs the back of control dogs. *p* values < 0.01 were regarded as statistically significant. Analyses were carried out in R language and environment for statistical computing (R version 3.5.1).

### Scanning electron microscopy

To further assess the structure of the HSs scanning electron microscopy (SEM) was performed from the plucked hairs of three dogs with BTS and four unaffected controls (S1 Table). Hairs were plucked using the same method as described for trichograms. A total of 35 hairs from the back and thigh from control dogs and 29 hairs from the same locations from dogs with BTS were examined. Prior to scanning four to six hairs per location were placed in their total length on 25 mm wide aluminum stubs. The hair samples were coated with gold using the EM ACE600 Sputter (Leica) and scanned with the scanning electron microscope Helios NanoLab 650 SEM (FEI, now Fisher Scientific). From each sample digital images were taken in different magnifications.

### RNA extraction and transcriptome sequencing (RNA-seq)

RNA for transcriptome sequencing was extracted from skin biopsies from the thigh of five dogs with BTS and seven control dogs (S1 Table). RNA extraction, library preparation and RNA-seq were performed as described in the detailed method description in S1 Text and a previous study [22]. The data are available in the European Nucleotide Archive (ENA), study accession PRJEB21761 and sample accessions SAMEA104393642-SAMEA104393658. (http://www.ebi.ac.uk/ena/data/view/PRJEB21761).

### Mapping to reference genome and differential gene expression analysis

All reads that passed quality control were mapped to the dog genome reference (Can.Fam3.1) by STAR aligner version 2.5.3a [23] as described in our previous study [22]. The read abundance was calculated using HTseq and a gff3 file (version 104) obtained from NCBI canFam3.1 annotation release [24]. We used the DESeq2 package [23] to read the HTseq count data and filter for low/non-expressed genes. DESeq2 applies a generalized linear model (GLM) to the normalized count data assuming a negative binomial distribution. Read counts for each gene were fit to a GLM with 2 factor design model (~sex + condition) where condition was the factor of interest with two states: control and BTS, and sex factor was used to control for the effect of sex. Transcripts were considered to be differentially expressed with a Benjamini and Hochberg false discovery rate (FDR) of < 0.05. A detailed method description is available in S1 Text.

### Proteomic analysis

Proteomic analysis was performed on fractured HSs of four dogs with BTS (Greyhounds, n = 3, Whippet, n = 1) and intact telogen HSs of four control dogs (Greyhounds, n = 3, Whippet = n = 1) plucked on the thighs (S1 Table). From each dog 0.25–0.29 mg of hair was cut in small pieces and washed successively three times with 50mM acetic acid and 20% ethanol before protein extraction as described [25] with some changes (see S1 Text). Extracted proteins were separated by SDS-PAGE and in-gel digested for nano liquid chromatography tandem mass spectrometry (nLC-MS/MS). Mass spectrometry data was processed with MaxQuant/Andromeda (version 1.5.4.1) to identify proteins at a 1% FDR and relatively quantify expression differences between control and BTS dog samples at a 5% FDR. Identical gel material, but without any protein sample loaded and processed in parallel, in order to assess keratin contaminations from the laboratory environment (S1 Text). The mass spectrometry proteomics data have been deposited to the ProteomeXchange Consortium via the PRIDE partner repository with the dataset identifier PXD012371.

### Whole genome sequencing and variant calling

We sequenced the whole genome of two affected and two normal haired Greyhounds (S1 Table) to investigate the underlying genetic etiology. For each dog, an Illumina PCR-free TruSeq fragment library with ~450–500 bp insert size was prepared. We collected ~260–337 million 2 x 150 bp paired-end reads on a NovaSeq 6000 instrument. The reads were mapped and variants were called using BWA-GATK best practices workflow with the following versions of the tools—BWA (version 0.7.15), samtools (version 1.9), picard tools (version 1.8), and GATK (version 3.6)[26–28]. The functional effects of the called variants were predicted using SnpEFF software (version 4.3T) [29] together with the NCBI annotation release 104 on CanFam 3.1. The sequence data were deposited under the study accession PRJEB16012 and sample accessions SAMEA4867932, SAMEA4848711, SAMEA4867933, and SAMEA4848712, at the European Nucleotide Archive. Additionally, we used 355 additional whole genome sequences as controls, which were either publicly available [30], produced during other projects of our group or contributed by members of the Dog Biomedical Variant Database Consortium; ENA study and sample accession numbers are available in S12 Table and a detailed method description can be found in S1 Text.

## Results

### Histological findings

**Fig 1:**
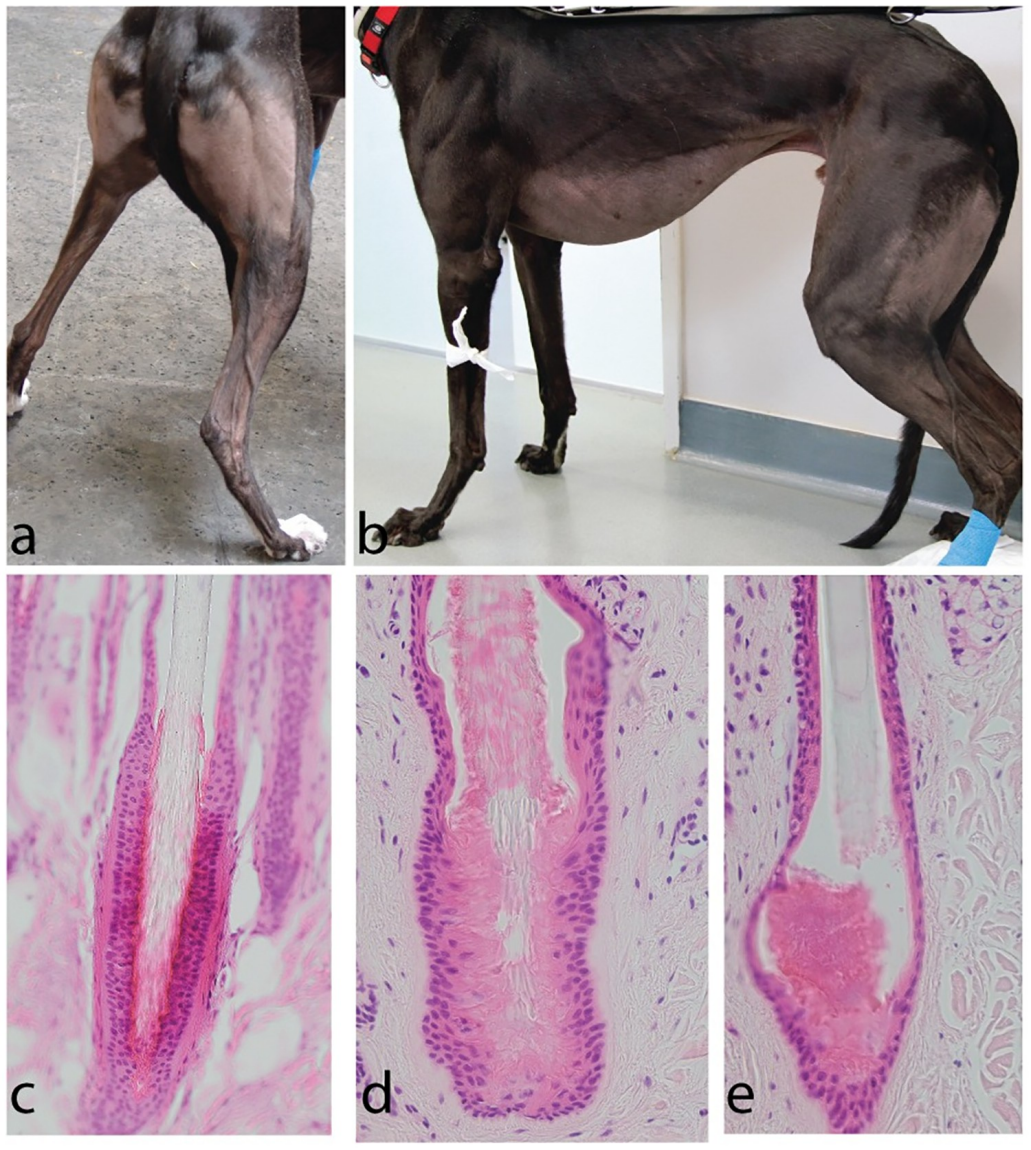
Clinical and histological phenotype of Greyhounds with BTS. (a) Affected dog with alopecia on the caudal and lateral thighs extending below the lateral hock area; (b) Affected dog with alopecia on the caudal and lateral thighs, the lateral hind leg down to the hock, the ventral abdomen and the ventral chest; (c) Normal telogen HF; (d) Telogen HF with an increased amount of trichilemmal keratin at the proximal end of the follicle. The more distal part of the HS is not anchored by trichilemmal keratin; (e) Horizontally broken telogen HF, which is not anchored by trichilemmal keratin and abundant keratin at the base of the follicle.

Histological findings were similar in all biopsies investigated and no clear difference between biopsies taken from alopecic skin and haired skin could be observed. In all biopsies, no matter if they were taken from alopecic skin of dogs with BTS or from haired skin of control dogs, HFs were present and of similar size. The follicular stage was not determinable in about 40% of the follicles due to the orientation of the biopsies, similar to previously published results in healthy dogs [31]. In the follicles where the cycle stage could be assessed, the anagen:telogen ratio was about 1:1 in both groups. Multiple HSs were present in the majority of the slightly dilated infundibula which contained otherwise a mildly increased amount of infundibular keratin in both groups. The diameters of the HSs did not differ between affected and control dogs. In one biopsy of one of the affected dogs one telogen HF was present which had an increased amount of trichilemmal keratin at the proximal end of the follicle and the more distal part of the HS was not anchored by trichilemmal keratin as is usually seen in club hairs (Fig 1d). In another biopsy of an affected dog the HS in one telogen HF was broken horizontally and not anchored by trichilemmal keratin as just described whereas the base of the HF was filled with abundant keratin (Fig 1e). The dermis was rather thin in both groups, which hampered correct embedding.

### Trichogram

**Fig 2:**
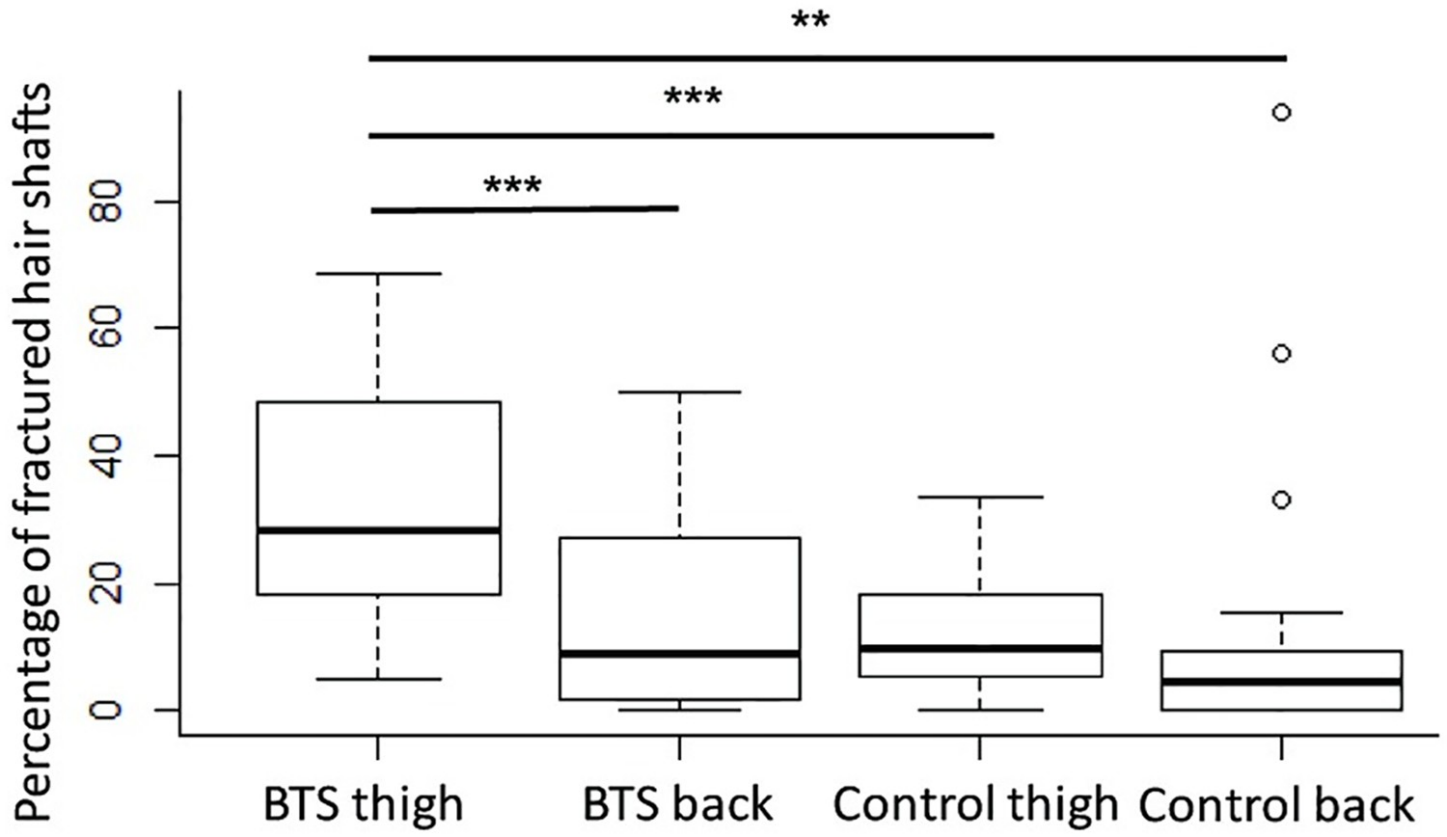
Boxplots illustrating the percentage of fractured hair shafts plucked from the thigh and back of affected and control dogs. The box includes the median of the data and the box edges are the 25^th^ and 75^th^ percentiles. The vertical size of the boxes displays the interquartile range (IQR); the whiskers represent the minimum and maximum values that do not exceed 1.5 x IQR from the median. ** *p* < 0.01 *** *p* < 0.001.

Trichogram analysis revealed a significantly higher number of HSs fractured on the proximal endin hairs plucked from the thigh of dogs with BTS. The number of fractured HSs was higher than from the back of dogs with BTS and from both locations in control dogs (S2 Table, Fig 2). However, clean transverse fractures at the proximal ends of the shafts were equally present on the back in both groups and on the thighs in control dogs (S2 Table, Fig 2). Unexpectedly, we found in two control dogs (C4 and C8) fractures in more than 50% of the plucked hairs from the back; visible as outliers in the boxplot. Other HS abnormalities, besides small melanin aggregates, depending on the colour of the dogs, were not observed in the trichograms.

### Scanning electron microscopy

**Fig 3:**
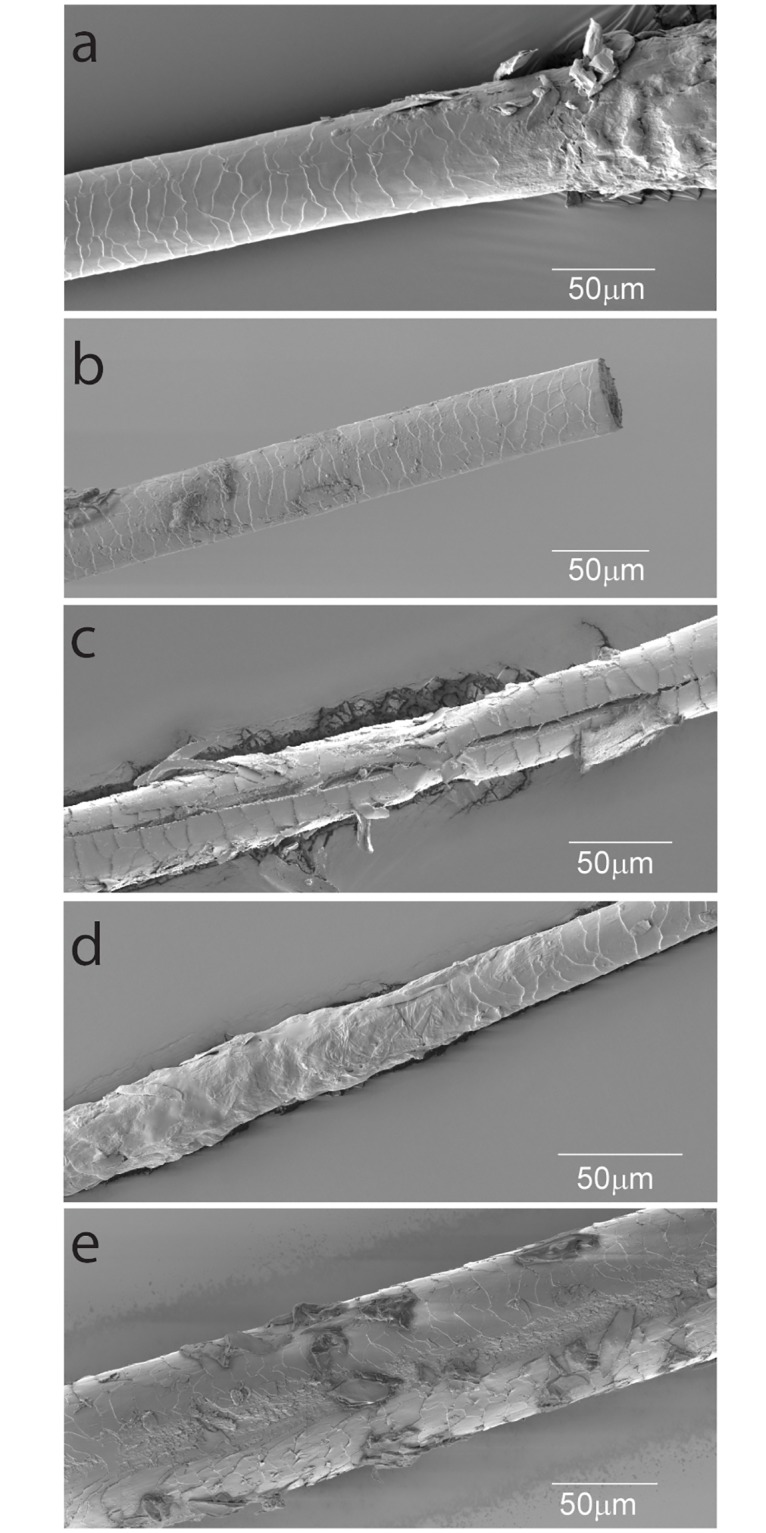
Structural abnormalities in hair fibers of Greyhounds identified by scanning electron microscopy. (a) Intact telogen hair root of a control Greyhound, (b-d) abnormal HSs from Greyhounds with BTS (b) Clean transverse fracture (trichoschisis); (c) Central longitudinal splitting (central trichoptilosis). (d) Irregular surface of the cuticle; (e) Longitudinal grooves.

SEM revealed several structural defects in HSs from Greyhounds with BTS and control dogs. The most frequently observed abnormality in control and affected dogs was a clean transverse fracture (trichoschisis) in the proximal part of the hair fiber (17/64 hairs). The percentage of fractured HSs was higher in Greyhounds with BTS (34%) as compared to controls (20%). The second common abnormality was a central longitudinal splitting (central trichoptilosis) of hair fibers. The splits varied in length and were between 50μm and 1.5mm long (7/64 hairs). The percentage was higher in dogs with BTS (17%) in comparison to 5.7% in controls. Rare findings were longitudinal grooves and an irregular outer contour of the HSs with fused cuticular cells (Fig 3) [32].

### Differentially expressed genes

Principal component analysis of the RNA-seq data revealed that BTS samples were successfully separated from control samples. In addition, clustering based on sex was found within the control group (S1 Fig). Cook’s distance revealed one outlier, which was excluded from the analysis (sample C3). It was a male Greyhound classified as unaffected control dog within our project. Histopathological re-evaluation of the skin biopsies revealed a low grade superficial perivascular and lymphocytic dermatitis, which could explain the large Cook’s distance and justified the exclusion of that sample. After filtering for low/non-expressed genes we analyzed a total of 22503 genes. We considered genes with an FDR of less than 0.05 as differentially expressed and discovered 442 differentially expressed genes. Of those, 74 genes (17%) were upregulated and 368 genes (83%) were downregulated in BTS affected dogs (S3 Table).

Numerous downregulated genes encode structural proteins such as keratins (23 genes) and KAPs (51 genes) of the HS and the IRS cuticle (S4 and S5 Tables).

Additionally, we identified 14 downregulated genes involved in HF and HS differentiation and variants in some of these genes are associated with HS disorders. Furthermore, genes encoding members of the BMP signaling pathway, *BMP2* and *BMP4*, were downregulated as well as some of their downstream acting genes (*FOXN1*, *HOXC13 and MSX2)*, which are known to play a role in HS formation (S6 Table).

### Proteomic analysis

**Fig 4:**
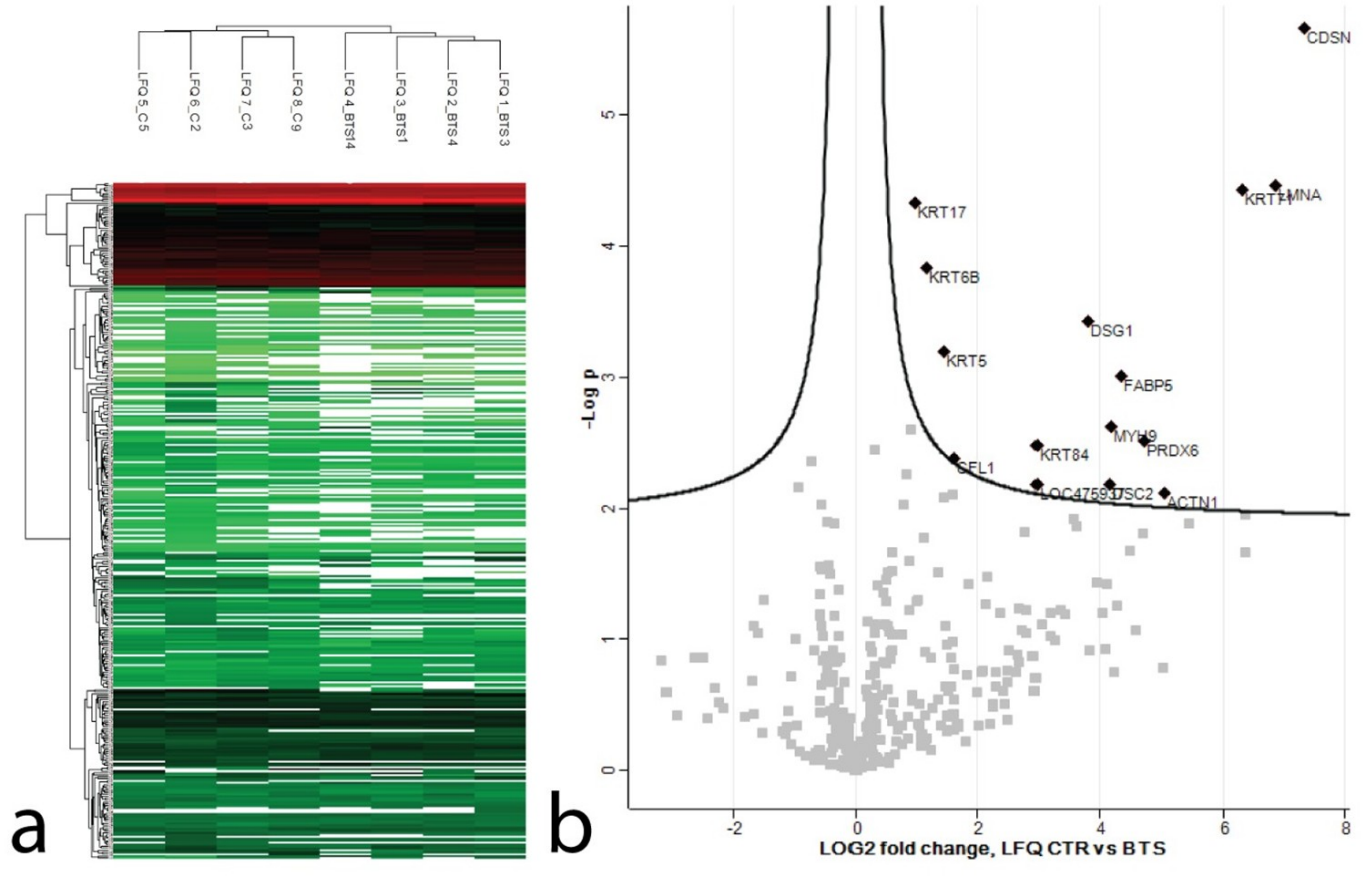
Canine hair proteins identified by nLC-MS/MS. (a) Hierarchical clustering of LFQ protein intensities. Highly abundant proteins are shown in red, low abundances in green, intermediate values in different shades of red and green and missing protein values are displayed in white. BTS affected dogs and control dogs form two distinct clusters. (b) Volcano plot representing the results of the T-test performed on LFQ intensities of control vs BTS. Differentially expressed proteins with a FDR < 0.05 and a log2 fold change cut-off value of 1 are labeled.

To confirm the results of the transcriptome analysis from whole skin and to further elucidate the structure of defect HSs of dogs with BTS, we performed proteomic profiling using a nano-scale liquid chromatographic tandem mass spectrometry (nLC-MS/MS) approach. Our aim was to identify differences in protein expression of fractured HSs from dogs with BTS (3 Greyhounds, 1 Whippet) as compared to intact telogen HSs from control dogs (3 Greyhounds, 1 Whippet). A total of 353 proteins were detected in HSs of dogs (S7 Table). Of those, 23 were identified as possible contaminants and 27 proteins were only identified by a modification site. After performing a Student’s T-test on log2-transformed label free quantitation (LFQ) values 15 significantly differentially expressed proteins between the control and BTS group with a log2 fold change above one were identified (Fig 4, S8 Table).

### Genetic analysis

To investigate possible genetic risk factors for BTS, we used whole genome sequencing data from two BTS affected and two unaffected Greyhounds as well as 355 dog genomes from diverse breeds. Applying a hard-filtering approach, we considered a recessive and a dominant mode of inheritance as well as a fixed risk allele in the Greyhound population (S1 Text). In the model of fully penetrant autosomal recessive mode of inheritance, 5 variants predicted to be protein-changing were exclusively found in homozygous state in the affected Greyhounds (S9 Table). One of them, a missense variant in the *CYP26C1* gene encoding an enzyme involved in the regulation of retinoic acid levels, was specific to Greyhounds in our sample. Filtering for variants consistent with a dominant mode of inheritance resulted in 64 protein-changing variants (S10 Table). These variants included a missense variant in the *BRD8* gene, encoding a thyroid-hormone receptor interacting protein. In both recessive and dominant scenarios, none of the protein changing variants was located in an obvious functional candidate gene or one of the genes that showed strong differential expression. Searching for variants that were present in the homozygous state in all available Greyhounds but not in any non-sighthound breed resulted in only one protein-changing variant (S11 Table), a missense variant in the *IGFBP5* gene; XM_847792.4:c.424C>T, p.(Arg142Cys). This variant was also present in a homozygous state in a Whippet, a Scottish Deerhound and three Sloughis, and present in a heterozygous state in a Saluki. We had no reliable information regarding a possible BTS phenotype in the other sequenced sighthounds. The twelve Greyhound transcriptomes obtained in this study also were from dogs that were homozygous for the alternate T-allele at this variant.

## Discussion

In our study we identified structural defects in the HSs as underlying cause for BTS in Greyhounds and related breeds. Transverse fractures and other structural defects in the proximal parts of the HSs have been discovered by trichograms and SEM. These findings are supported by the histological analysis and the results of the transcriptomic and proteomic profiling where genes and proteins important for differentiation of the IRS and the assembly of a proper HS were downregulated.

Our results contradict the description in a veterinary textbook in which BTS is reported as pattern baldness [33]. The histological characteristic of this type of alopecia is the miniaturization of HFs resulting in thin to minuscule HSs, which cannot be appreciated in skin biopsies from dogs with BTS when compared to biopsies from the same body location of haired control dogs. Histologically, we saw in both affected and control dogs mildly dilated HF infundibula with numerous HSs of a similar diameter in the lumen. In addition, we found a similar percentage of anagen and telogen follicles, as has been described in another study where dogs with a short and fine hair coat have been histologically evaluated [34]. In the only so far existing histological study of BTS a dilatation of follicular infundibula filled with keratin and hair has also been described [21]. However, in this study the authors did not compare skin biopsies of alopecic Greyhounds with control dogs of other breeds. Thus, we assume that a mild dilation of the infundibulum is a breed-specific feature in the caudal thighs of Greyhounds and other sighthound breeds. In addition, the presence of normal sized HSs within the infundibulum does neither support a hair cycle arrest, in which HSs are not produced any longer, nor a pattern baldness in which the HSs should be thinner than in control dogs.

Using light and SEM we found a significantly higher percentage of HS defects on the caudal thighs of the affected Greyhounds and Whippets as compared to the back of the same dogs and the back and the thighs of control dogs. HS defects can be attributed to mechanical, physical, and chemical injury or may have an underlying inherited cause [35]. Damage caused by environmental insults is mostly seen in the exposed distal parts of the HSs, whereas inherited defects can also occur in the more proximal parts of the HS which are protected within the HF [36]. The HS fractures in our study group were exclusively seen in the proximal part close to the hair root, suggesting an inherited defect. The fact that the percentage of fractured HSs was significantly higher on the thighs of affected dogs as compared to the back of the same dogs might be explained by different hair types on different body locations. In addition, the severity of structural defects may change due to yet unknown circumstances, which might explain that BTS is waxing and waning in some dogs and that some dogs recover completely.

The most frequently observed defects were clean transverse fractures (central trichoschisis) and central longitudinal splitting (central trichoptilosis). In addition, we found HSs with longitudinal grooves. These HS defects are known in humans and causative genetic variations have been identified in some of them [36–39]. Furthermore, we identified HSs with an irregular outer contour and fused cuticular cells, a structural defect which has not been described so far. Central trichoptilosis and HSs with longitudinal grooves have been described in humans in association with ectodermal dysplasia [40]. Ectodermal dysplasia has been defined as an abnormal development of at least two different tissues or organs of ectodermal origin [41–45]. Since no other tissue defect has been identified in sighthounds with BTS the HS defects cannot be attributed to ectodermal dysplasia in the strict meaning. However, several other conditions in humans exist in which the HS is the only ectodermal appendage affected and in some of them an underlying genetic defect has been identified. Described alterations of the HS associated with hypotrichosis or alopecia range from thin, brittle, or coarse HSs to severe structural defects within the HSs [36, 41, 43, 44]. The far best described HS defect in humans is monilethrix. In this nonsyndromic hair disorder the HS has intermittent constrictions separating elliptical nodes of normal thickness. Most of the cases of monilethrix are caused by variants in keratin genes, but it is also described with variants in the desmoglein 4 gene (*DSG4*). [41, 44, 46–48]. Trichothiodystrophy (TTD) is another heterogeneous group of autosomal recessive disorders in which clean transverse fractures of the HSs, similar to those seen in our study are the morphological hallmark. Furthermore, an irregular hair surface, HS diameter and a decreased cuticle layer may be present. The underlying cause of TTD is a defective synthesis of high-sulfur matrix proteins (reviewed in [36, 39, 41]. In all so far described HS disorders in humans the HSs break easily with trauma caused by environmental insults or mechanical stress. Interestingly, also in humans hair loss may occur periodically and intermittent hair loss associated with infections has been described. As suggested before, trauma or subclinical infections may also explain the waxing and waning of the alopecia seen in dogs with BTS.

In order to investigate the molecular pathomechanisms involved in BTS we performed RNA-seq of whole skin biopsies to define differences in gene expression levels between affected and control dogs. A total of 23 genes encoding HF and HS specific keratins and 51 genes encoding KAPs was downregulated in the skin of dogs with BTS (S4 and S5 Tables). Both keratins and KAPs are the major proteins of the trichocytes and essential for a mechanically stable and compact hair fiber [12, 49]. Thus, these results suggest that the HS defects identified in BTS are the consequence of the downregulation of several genes encoding for proteins which are crucial for the mechanical stability and integrity of HS [3]. Interestingly, the keratin genes *KRT81*, *KRT83*, *KRT86*, as well as the desmosomal cadherin-coding gene DSG4, were downregulated in dogs with BTS. Variants within these genes have been identified in monilethrix but the expression has not been investigated on the mRNA level [47, 48, 50]. Vice versa genetic analysis in our cases did not reveal variants within these genes. The keratin gene family is largely, but not perfectly conserved between humans and dogs [51].

Beside the downregulation of keratins and KAPs we observed downregulation of 14 other genes involved in HS differentiation and assembly of the HS (S6 Table). Amongst those, we identified a significant downregulation of *BMP2* and *BMP4* as well as the downstream acting regulators of HS differentiation *MSX2*, *FOXN1* and *HOXC13* in skin biopsies of dogs with BTS. It has been shown in mice that ablation of Bmp signaling downregulates the expression of several transcription factors such as Msx2, Fox1 and Hoxc13 resulting in a reduced expression of acidic hair keratins and trichohyalin leading to a severely impaired HS differentiation [52]. Other downregulated genes, namely *PADI3* and *TCHH* are associated with uncombable hair syndrome in humans [53] and *S100A3* has been connected with an age-dependent damage of the hair cuticle [54, 55]. Furthermore, it is well known that desmosomal cadherins such as the downregulated *DSC2* and *DSG4* are crucial to crosslink the IFs and the KAPs and are thus essential for a mechanically stable and compact hair fiber [8].

To further elucidate the origin of the HS defects, we performed proteomic analysis of fractured HSs from dogs with BTS and intact telogen HSs from control dogs. Proteomic analysis revealed differential protein expression of 15 proteins. Among others, we found a decreased protein expression of keratin 71 (KRT71), desmoglein 1 (DSG1), corneodesmosin (CDSN), and desmocollin 2 (DSC2). In line with the downregulation of *KRT71* on the mRNA level is the decreased protein expression of KRT71 (Table 1). This suggests that KRT71 plays an important role in the pathomechanism of BTS, although no sequence variant in the gene has been identified in dogs with BTS. In mice and humans KRT71 protein is expressed in the IRS [56, 57], but the localization of KRT71 expression has not been shown in dogs yet. KRT71 is crucial for hair texture and different variants in the *KRT71* gene result in wooly or curly hair phenotypes seen in *caracul* mice, *rex* rats, Devon Rex cats and many curly coated dogs [58–60]. The autosomal dominant *Rex* variant in rats leads to hair loss in the homozygous state [61]. In reduced coat 3 (Rco3) mice carrying an autosomal recessive variant in *Krt71* progressive alopecia develops due to alterations in the keratinization of the IRS and subsequent structural defects in the HSs [62]. In the hairless Sphynx cat a variant in *KRT71* in a homozygous state or compound heterozygous with the curly (re) allele of the closely related Devon Rex cats leads to abnormalities of IRS and misshapen HSs [63]. Furthermore, a missense variant in *KRT71* causes the autosomal dominant wooly hair and hypotrichosis phenotype in humans and in cells transfected with the mutant KRT71 protein a disruption of IF formation was observed [64]. Since KRT71 is known to be expressed in the IRS in mice and humans, and the expression in HSs has not been described yet, further investigation of KRT71 protein expression in the HSs of dogs is needed.

**Table 1:** Differential gene and protein expression in hair shafts and skin of dogs with BTS vs controls.

	Protein expression		Gene expression	
Gene	FDR	log2FC BTS vs Ctrl	FDR	log2FC BTS vs Ctrl
KRT71	2.13E-02	-6.32	1.07E-10	-1.51
DSC2	2.90E-02	-4.15	3.01E-02	-0.52

Differential protein expression was investigated by proteomic profiling of fractured hair shafts from dogs with BTS vs intact telogen hair shafts from controls. Differential gene expression was studied using RNA-seq of whole skin biopsies from dogs with BTS and controls.

We further found a downregulation of *DSC2* on the mRNA level in skin biopsies and a decreased protein expression in the defect HSs of dogs with BTS (Table 1).

Dsc2 is expressed in the medulla of HSs in mice, specifically between the vertically assembled medulla cells and between cortex and medulla cells and it is assumed that DSC2 plays an essential role in the assembly of the hair medulla [65, 66]. Interestingly, in mice the expression of Dsc2 in medulla has been associated with the expression of Foxn1, a transcription factor downstream of Bmp, which is also downregulated in dogs with BTS [67]. Foxn1 deficient mice (*nude* mice) display HS defects which lead to a fragmentation of HSs and subsequent alopecia in mutant mice. The reduced transcription of Dsc2 in HSs and low abundance of Dsc2 protein in medulla cells adjacent to cortex cells as a consequence of Foxn1 deficiency result in improper assembly of medulla cells and therefore may be the reason for fragile HSs in *nude* mice [66].

Two other proteins of interest which were decreased in fractured HSs are CDSN and DSG1. CDSN is expressed in the IRS and in squamous epithelium of skin and variants in *CDSN* in humans are associated with hypotrichosis simplex of the scalp. Patients with hypotrichosis simplex develop progressive hair loss starting in late childhood. As possible pathomechanism for the alopecia, the authors discuss a loss of cohesion within the IRS or protein aggregates of CDSN [68]. DSG1 is an important protein of desmosomes which anchor IFs within their matrix and assemble macrofibris [3, 69]. The reduced protein may impair tissue integrity of HSs and assembly of cortex and medulla cells and thus result in defect HSs in dogs with BTS.

A limitation of our study is the fact that transcriptional and proteomic profiling has not always been performed with the same dogs (S1 Table) and thus comparison of the results may have some weaknesses. However, the finding that even in whole skin biopsies genes coding for HS proteins are significantly downregulated in biopsies from dogs with BTS and that two of these are even differentially expressed on the protein level strongly suggests a correlation and biological significance. We are also aware that protein expression might be influenced by the missing very proximal part of the fractured HS, which is present in the intact telogen HSs and we face the risk that the lower expression of some proteins may be interpreted wrongly.

In our genetic study we did not identify a compelling candidate genetic variant that co-segregated with the BTS phenotype. This is most likely due to a complex mode of inheritance, which would not have been tractable with our simple hard filtering approach in only two cases and two controls. However, we found a missense variant in the *IGFBP5* gene that was apparently fixed in the homozygous state in all available Greyhounds and some other sighthounds and absent or rare in other breeds. Insulin-like growth factor binding proteins (IGFBPs) are secreted proteins that are able to bind and antagonistically modulate effects of insulin-like growth factors. *IGFBP5* has an influence on numerous biological processes, but mainly its potential to enhance or suppress cell proliferation has been investigated. *Igfbp5* mRNA is expressed in the dermal papilla of murine anagen HFs as well as in dermal cells and the subcutis. The expression of *Igfbp5* in the dermal papilla decreases at the beginning of catagen, while the expression in the dermis is maintained during the resting phase of the hair cycle [70]. Furthermore, *Igfbp5* expression correlated with the bent structure of zigzag hair, one of the four hair types of mouse pelage. In transgenic mice, ectopic *Igfbp5* expression led to thin and curved hair [71]. There is a lack of literature about *IGFBP5* expression in dogs, however, given the knowledge in mice, it is likely that *IGFBP5* also has an influence on HS differentiation in dogs and we cannot exclude that the expression pattern of *IGFBP5* in the HFs of the thighs and the ventral aspects of the dogs differs from the HFs of the back. Whether the identified genetic variant has an impact on protein function and whether it represents a predisposing risk factor for BTS remains however to be determined.

Taken together, we have shown that BTS is caused by structural HS defects which are associated with the downregulation of genes and proteins essential for HS differentiation and HS assembly. The underlying genetic defect has not yet been identified and we suggest a complex mode of inheritance. Our data add important knowledge to further understand the molecular mechanisms of hair shaft formation and alopecia in dogs.

## Supporting information

S1 Text Detailed material and methods of transcriptomic, proteomic and genetic analysis. (DOCX)

S1 Table List of dogs used in our experiments including condition, breed, sex, age and method applied. BTS, bald thigh syndrome; C, control; m, male; mc, male castrated; f, female; fc, female castrated; SEM, scanning electron microscopy; WGS, whole genome sequencing.
(XLSX)

S2 Table Results of trichogram analysis indicating number and percentage of fractured hair shafts plucked from the thigh and the back of dogs affected with bald thigh syndrome (BTS) and control dogs (C). * Hairs were plucked from the hypotrichotic area on the border alopecia/haired.
(XLSX)

S3 Table List of differentially expressed genes with a false discovery rate (FDR) of less than <0.05. Sorted by log2 fold change. ID, gene identifier of the Entrez Gene database of NCBI; BaseMean, mean of normalized read counts across all samples; LfcSE, standard error of the log2 Fold Change; Stat, the log2 fold change divided by lfcSE; FDR, false discovery rate, refers to Benjamini-Hochberg adjusted p- value.
(XLSX)

S4 Table List of downregulated genes coding for keratin genes and their expression in the HS and HF. ID, gene identifier of the Entrez Gene database of NCBI; Log2FC, log2 fold change; FDR, False discovery rate, refers to Benjamini-Hochberg adjusted p- value. Keratin expression in humans and sheep as reviewed in Harland, D.P. and Plowman, J.E. [3].
(XLSX)

S5 Table List of downregulated genes coding for keratin-associated proteins. ID, gene identifier of the Entrez Gene database of NCBI.; Log2FC, log2 fold change; FDR, false discovery rate, refers to Benjamini-Hochberg adjusted p- value.
(XLSX)

S6 Table List of downregulated genes important for HF differentiation and HS formation, their tissue expression and disorders associated with variants in these genes including references. ID, gene identifier of the Entrez Gene database of NCBI; Log2FC, log2 fold change; FDR, false discovery rate, refers to Benjamini-Hochberg adjusted p- value; IRS, inner root sheath; HF, hair follicle; ORS, outer root sheath; DP, dermal papilla. [50, 52–55, 65, 66, 72–83].
(XLSX)

S7 Table List of proteins identified by nLC-MS/MS in hair shafts of dogs. iBAQ, intensity based absolute quantification; LFQ, label free quantitation; top3, median normalized intensities of the three most intense peptides.
(XLSX)

S8 Table Results of Student’s T-test on log2-transformed label free quantitation (LFQ) protein intensities. Missing protein intensities were replaced sample wise by imputing a random number from the low end of the log2-transformed LFQ distribution; test differences were calculated on the median LFQ intensities (log2 transformed) and *p* values (-log) were corrected for multiple testing by a permutation based approach to estimate a 5% false discovery rate (*q* values); significant test results are marked by “+” in the “significant C vs BTS” column, if a protein in the control group is significantly higher expressed than in BTS and gene names of significantly differentially expressed proteins are highlighted in bold characters.
(XLSX)

S9 Table Protein-changing variants homozygous in cases (BTS3, BTS19), heterozygous or absent in control Greyhounds and controls from other breeds. 0 stands for the wild type allele, 1 for the variant allele. Dog GY432 was excluded due to unknown phenotype.
(XLSX)

S10 Table Protein-changing variants hetero- or homozygous in cases, absent in control Greyhounds and controls from other breeds. 0 stands for the wild type allele, 1 for the variant allele. Dog GY432 was excluded due to unknown phenotype.
(XLSX)

S11 Table Protein-changing variants homozygous in all Greyhounds (N = 5), heterozygous or absent in other breeds. 0 stands for the wild type allele, 1 for the variant allele.
(XLSX)

S12 Table Sample and study accession numbers of whole genome sequencing data including sample IDs and breed information of dogs used in the genetic analysis. (XLSX)

S1 Fig Principal component analysis of the samples in the first two component space. Samples are plotted across the two most variable components (PC1 and PC2) and sample clustering is based on condition.
(TIF)
