# Supplementary material for: Bald thigh syndrome in sighthounds—Revisiting the cause of a well-known disease
Source: PLoS One. 2019 Feb 22;14(2):e0212645. doi: 10.1371/journal.pone.0212645 (PMC6386255; doi:10.1371/journal.pone.0212645)
Supplement: S1 Text — (DOCX) [file pone.0212645.s001.docx]

# S1 Text. Detailed Material and Methods

## **RNA extraction and transcriptome sequencing (RNA-seq)**

Prior to RNA extraction the skin biopsies from five affected and seven control dogs were homogenized mechanically with the TissueLyser II device from Qiagen. Total RNA was extracted from the homogenized tissue using the RNeasy Fibrous Tissue Mini Kit (74704; Qiagen) according to the manufacturer’s instructions. RNA quality was assessed with a FragmentAnalyzer (Advanced Analytical). From each biopsy 1 µg of high quality RNA (RNA integrity number: RIN > 9) was used for library preparation (TruSeq Stranded mRNA, Illumina). Twelve multiplexed mRNA libraries were sequenced on one lane using the Illumina HiSeq3000 with 2x150 bp paired-end sequencing cycles. Twenty million read pairs per stranded library were collected on average. The Illumina BCL output files with base calls and qualities were converted into FASTQ file format and demultiplexed. The data are available in the European Nucleotide Archive (ENA), study accession PRJEB21761 and sample accessions SAMEA104393642, SAMEA104393643, SAMEA104393644, SAMEA104393645, SAMEA104393646, SAMEA104393647, SAMEA104393648, SAMEA104393649, SAMEA104393650, SAMEA104393651, SAMEA104393652, SAMEA104393653, SAMEA104393654, SAMEA104393655, SAMEA104393656, SAMEA104393657, SAMEA104393658. (http://www.ebi.ac.uk/ena/data/view/PRJEB21761).

## **Mapping to reference genome**

All reads that passed quality control were mapped to the dog genome reference (Can.Fam3.1) by STAR aligner version 2.5.3a (1). Reads were aligned using the following parameters: --outFilterType BySJout --outFilterMultimapNmax 50 --alignSJoverhangMin 1 --outFilterMismatchNmax 2 --outFilterMismatchNoverLmax 0.04 -- alignIntronMin 20 --alignIntronMax 1000000 --alignMatesGapMax 1000000. The alignment of RNA-seq reads from each sample was summarized by the number of uniquely mapped reads per sample including both singleton and both-ends mapped and number of splice alignments per sample. The read abundance was calculated using HTseq and a NCBI transcript database (version 105) derived from the CanFam3.1 dog genome assembly (2)

## **Differential expression**

We used the DESeq2 package(1) to read the HTseq count data and filter for low/non-expressed genes where the count is zero in all samples and a single count in one sample. The count data were subjected to a regularized-logarithm transformation and a principal component analysis (PCA) was performed to visualize the clustering of the case and control groups. Following PCA analysis we used DESeq2 v.1.6.3 to assess differential expression between groups. DESeq2 applies a generalized linear model (GLM) to count data assuming a negative binomial distribution. Read counts for each gene were fit to a GLM with design model (~sex + condition) where condition was the factor of interest with two states: control and BTS. Transcripts were considered to be differentially expressed with a Benjamini and Hochberg false discovery rate (FDR) of < 0.05. Further diagnostic tests, such as the Cook’s distance, were applied to detect outliers and assess good quality of the data.

## **Proteomic analysis**

Proteomic analysis was performed on fractured hair shafts of four dogs with BTS (Greyhounds, n=3, Whippet, n=1) and intact telogen HSs of four control dogs (Greyhounds, n=3, Whippet= n=1) plucked on the thighs (S1 Table). Intact telogen HSs and fractured HSs were carefully selected using a Keyence VHX-6000 digital microscope. From each dog 0.25-0.29 mg of hair was cut in small pieces and washed successively three times with 50mM acetic acid and 20% ethanol before protein extraction as as described by Wong et al. with some changes (3). Hairs were lysed by vortexing in 150µL 0.2M NaOH, 1% SDS, 2% beta-mercaptoethanol and 0.01M EDTA for 30 minutes at 90°C. The supernatant after centrifugation was kept and two additional extractions were done with 150µL lyses buffer by vortexing for 30 minutes at room temperature. The three supernatants were pooled and proteins precipitated with five volumes of acetone at -20°C overnight. After centrifugation at 16000 g for 10 min, the dry pellet was resuspended in 30µL Laemmli buffer/ 40mM DTT, boiled for 5 minutes at 95°C and loaded on a 12% SDS-PAGE gel. The gel was developed for about 1.5 cm, stained with Coomassie blue, and each lane was cut into five bands of equal size. In parallel, two gels with the neat Laemmli buffer were processed the same way as a background sample (blank). Gel bands were cut into small cubes, which were transferred to 1.5mL polypropylene reaction vials and stored under 100µL 20% (v/v) ethanol at 4°C until digestion.

Proteins were in-gel digested as described in (4) . An aliquot of 5µL from each digest was analysed on a nano liquid chromatography tandem mass spectrometry (nLC-MS/MS) instrument consisting of an EASY-nLC 1000 chromatograph coupled to a QExactive HF mass spectrometer (ThermoFisher Scientific). Peptides were trapped on an Acclaim C18 PepMap100 pre-column (5μm, 100 Å, 300μm x 5mm, ThermoFisher Scientific, Reinach, Switzerland) and separated by backflush on a C18 column (3μm, 100 Å, 75μm x 15 cm, Nikkyo Technos, Tokyo, Japan) by applying a 40 min gradient of 5% acetonitrile to 40% in water, 0.1% formic acid, at a flow rate of 350 nl/min. Peptides of m/z 400-1400 were detected at a resolution of 60’000 (at m/z 250) with an automatic gain control (AGC) target of 1E06 and maximum ion injection time of 50 ms. A top fifteen data dependent method for precursor ion fragmentation was applied with the following settings: precursor ion isolation width of 1.6 m/z, resolution 15’000, AGC of 1E05, maximum ion time of 110 ms, charge inclusion of 2+ to 7+ ions, peptide match on, normalized HCD fragmentation energy of 28%, and dynamic exclusion for 20 sec.

Mass spectrometry data were processed with MaxQuant/Andromeda version 1.5.4.1. The blank gel pieces were processed first against a SwissProt human sequence database (release 2017_12) with the following parameters: Mass error tolerance for parent ions of 10 ppm in the first, 4.5 ppm in the second round and fragment ions of 20 ppm; trypsin cleavage mode (no P rule) with 3 missed cleavages; static carbamidomethylation on Cys; variable oxidation on Met; deamidation on Asn and Gln; acetylation of protein N termini; match between runs activated with fraction numbering used. Based on reversed database peptide spectrum matches a 1% false discovery rate (FDR) was set for acceptance of peptide spectrum matches (PSM), peptides, and proteins. Additionally, two distinct peptides were required for the acceptance of a protein identification. All identified proteins together with porcine trypsin were used to create a contaminants database. Mass spectrometry data from the dog hair samples were then also interpreted with MaxQuant (same parameters) searching against the forward and reversed UniprotKB database of *Canis Lupus Familiaris* (release 2018_08) with added keratin sequences from an own dog genome sequencing project and the contaminants database (5)

The MaxQuant normalized label-free protein group intensity (LFQ) values were then used for statistical evaluation of differentially expressed proteins between the control and BTS group by Student’s T-test. LFQ values were log2-transformed, missing protein intensities were replaced sample wise by imputing a random number from the low end of the log2-transformed LFQ distribution. This imputation allowed for the calculation of *p*-values. The true test differences for proteins with missing values in one group were then calculated on the effective median intensities. *p*-values were corrected for multiple testing by a permutation based approach to estimate a 5% false discovery (q values), and a log2 fold change of one was set for acceptance of a significant change.

## **Genetic analysis**

To investigate a possible genetic factor in bald thigh syndrome, we sequenced the genomes of two Greyhounds affected with bald thigh syndrome (BTS3, BTS19) and two unrelated control Greyhounds (C20, C2) at ~31-40 x coverage. SNVs and short indels were called with respect to the canine reference genome CanFam 3.1 using BWA-GATK pipeline outlined as follows. BWA (version 0.7.15) was used for mapping to reference genome with default parameters and the output sam file was co-ordinate sorted and converted bam using samtools (version 1.9). The duplicate reads were marked with picard tools. (version 1.8). GATK was also used for base quality recalibration with canine dbsnp version 139 data as training set. Putative SNVs were identified in each of the 359 samples individually using GATK HaplotypeCaller in gVCF mode (6). Subsequently all sample gVCF files were joined using Broad GenotypeGVCFs walker (-stand_call_conf 30.0). Filtering was performed using the variant filtration module of GATK using the following standard filters: SNPs: Quality by Depth: QD < 2.0; Mapping quality: MQ < 40.0; Strand filter: FS > 60.0; MappingQualityRankSum: MQRankSum < -12.5; ReadPosRankSum < -8.0. INDELs: Quality by Depth: QD < 2.0; Strand filter: FS > 200.0. The filtered VCF file was annotated using SnpEff software version 4.3T and NCBI CanFam3.1 gff3 annotation file. The SnpEff annotated VCF file was compared to 355 control dog genomes from diverse breeds which were either publicly available (7), produced during other projects of our group or contributed by members of the Dog Biomedical Variant Database Consortium. We searched for candidate causative genetic variants considering three different possible scenarios: First, a recessive mode of inheritance, second, a dominant mode of inheritance, and third, a fixation of the variant in the Greyhound breed (Table 1). Among the control dogs, there was one Whippet (WH083), one Saluki (SL006), one Scottish Deerhound (SRS932150), three Sloughis (SG005, SG006, SG008) as well as one Greyhound with unknown phenotype (GY432), all of which were excluded during the filtering steps (except for the third scenario, where GY432 was included). By filtering variants consistent with a recessive mode of inheritance, we found 959 variants that were homozygous in the two affected Greyhounds and heterozygous or absent in control Greyhounds as well as other control dogs. We prioritized protein-changing variants (SnpEFF predicition) and found five such variants. Among those, we did not identify any variant in an obvious candidate gene. One of the variants, a missense variant in the *CYP26C1* gene encoding an enzyme involved in the regulation of retinoic acid levels, was private to Greyhounds. Assuming a dominant mode of inheritance, our automated pipeline detected 2201 variants that were hetero- or homozygous in the two affected Greyhounds and absent in control Greyhounds and other control dogs. Among the variants, 64 were protein-changing, but none of the protein-changing variants was located in an obvious candidate gene. These variants also included a missense variant in the *BRD8* gene, encoding a thyroid-hormone receptor interacting protein. To look for a potentially fixed risk allele in the breed, we filtered for variants that were present in the homozygous state in all Greyhounds and absent or present in the heterozygous state in the remaining 348 control dogs. This resulted in only 1 protein-changing variant, a missense variant in the *IGFBP5* gene; XM_847792.4:c.424C>T, p.(Arg142Cys). *IGFBP5* encodes the insulin-like growth factor binding protein 5, which is expressed in the hair follicle. The identified variant was also present in a homozygous state in the Whippet, the Scottish Deerhound and the three Sloughis, and present in a heterozygous state in the Saluki. By visual analysis using the integrative genomics viewer (8), we also found homozygous variant genotypes for the c.424C>T variant in the 12 transcriptomes from Greyhounds.

Table 1: number of SNVs and short indels detected after different filtering steps under the assumption of a recessive (1), or dominant (2) mode of inheritance, or a fixed risk-allele in Greyhounds.

| scenario | filtering step | # variants |
| --- | --- | --- |
| 1 | homozygous in cases; heterozygous or absent in control greyhounds and controls from other breeds | 959 |
|  | protein-changing homozygous variants in 1 | 5 |
|  | protein-changing private to Greyhounds in 1 | 1 |
| 2 | hetero- or homozygous in cases; absent in control greyhounds and controls from other breeds | 2201 |
|  | protein-changing heterozygous variants in 2 | 64 |
| 3 | homozygous variants in all Greyhounds; heterozygous or absent in other breeds | 8 |
|  | protein-changing homozygous variants in 3 | 1 |

# **References**

1. Love MI, Huber WA, S. . Moderated estimation of fold change and dispersion for RNA-seq data with DESeq2. Genome Biology. 2014;15(12):550.

2. Anders S, Pyl P, Huber W. HTSeq--a Python framework to work with high-throughput sequencing data. Bioinformatics. 2015;31(2):166-9.

3. Wong SY, Lee CC, Ashrafzadeh A, Junit SM, Abrahim N, Hashim OH. A High-Yield Two-Hour Protocol for Extraction of Human Hair Shaft Proteins. PLoS One. 2016;11(10):e0164993.

4. Gunasekera K, Wuthrich D, Braga-Lagache S, Heller M, Ochsenreiter T. Proteome remodelling during development from blood to insect-form Trypanosoma brucei quantified by SILAC and mass spectrometry. BMC genomics. 2012;13:556.

5. Balmer P, Bauer A, Pujar S, McGarvey KM, Welle M, Galichet A, et al. A curated catalog of canine and equine keratin genes. PLoS One. 2017;12(8):e0180359-e.

6. Van der Auwera GA, Carneiro MO, Hartl C, Poplin R, Del Angel G, Levy-Moonshine A, et al. From FastQ data to high confidence variant calls: the Genome Analysis Toolkit best practices pipeline. Current protocols in bioinformatics. 2013;43(1110):11.0.1-.0.33.

7. Bai B, Zhao W-M, Tang B-X, Wang Y-Q, Wang L, Zhang Z, et al. DoGSD: the dog and wolf genome SNP database. Nucleic acids research. 2015;43(Database issue):D777-D83.

8. Robinson JT, Thorvaldsdóttir H, Winckler W, Guttman M, Lander ES, Getz G, et al. Integrative genomics viewer. Nature biotechnology. 2011;29(1):24-6.
