# Supplementary figures and images for: Bald thigh syndrome in sighthounds—Revisiting the cause of a well-known disease
Source: PLoS One. 2019 Feb 22;14(2):e0212645. doi: 10.1371/journal.pone.0212645 (PMC6386255; doi:10.1371/journal.pone.0212645)

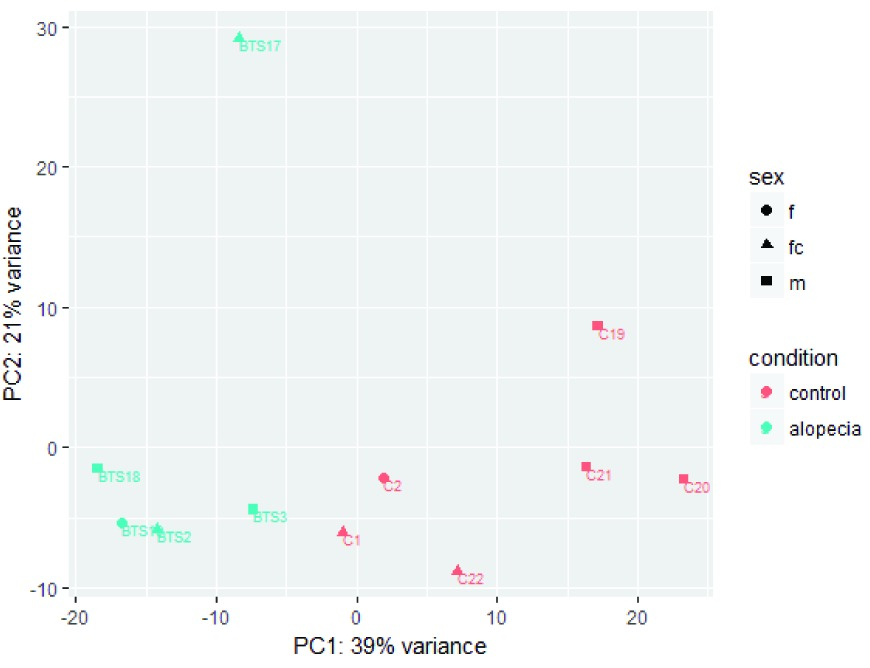

Supplement: S1 Fig — Samples are plotted across the two most variable components (PC1 and PC2) and sample clustering is based on condition. (TIF) [file pone.0212645.s014.tif]
